# An Automated Reverse-Bias Second-Breakdown Transistor Tester

**DOI:** 10.6028/jres.096.016

**Published:** 1991

**Authors:** David Berning

**Affiliations:** National Institute of Standards and Technology, Gaithersburg, MD 20899

**Keywords:** automated testing, clamp overshoot, crowbar, fast switching, nondestructive, protection circuit, reversebias, safe-operating area, second breakdown, transistor

## Abstract

An automated instrument is described for generating curves for the reverse-bias, safe-operating area of transistors nondestructively. A new technique for detecting second breakdown that makes automation possible is highlighted. Methods to reduce stress to the device under test are discussed, as are several other innovations that enhance automation. Measurements using the tester are described, and limitations on nondestructive testability are discussed.

## 1. Introduction

High-voltage, power-switching transistors are used in a wide variety of applications including such diverse fields as power conversion, motion control, and electronic ignition. A critical element for specifying the performance and reliability of these transistors is their turn-off capability. Most applications using high-voltage switching transistors require the transistor to turn off from a state of high-current conduction at low voltage with a load circuit that may be somewhat inductive. Often during turnoff, the voltage rises to a high value before the current begins to fall, and there is a period of time when the transistor experiences a very high level of peak power dissipation. If a particular combination of current and voltage exceeds the switching capability of the transistor, it may enter second breakdown and be destroyed. It is desirable to have test apparatus that can test the transistors by simulating conditions that are typical of actual usage in circuits, and it is especially desirable that the testing be nondestructive so that one transistor can be used to determine a safe operating area curve.

Equipment for testing the turn-off switching capability of high-voltage switching transistors has been previously described [1–3], and various data obtained from such testers have been discussed [4, 5]. These previous testers are difficult to use because each test requires multiple set-up steps and once a breakdown is observed, subjective interpretation is required. This paper describes a new tester that was developed to automate the process of making measurements of reverse-bias, safe-operating area. The tester was designed as a standalone instrument that can be used manually, or with a computer that has an IEEE-488 interface controller. This paper concentrates on the special techniques required to automate the measurement of reverse-bias safe operating area. The circuit details, including a complete set of schematic drawings, are published elsewhere [6].

## 2. The Automated Tester

**Figure 1:**
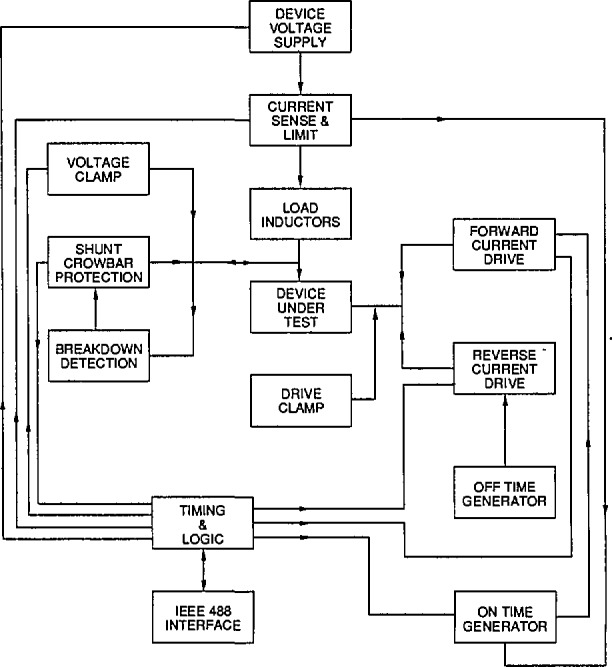
Block diagram of the tester.

Figure 1 is a block diagram of the tester. This tester, like others, works by connecting the device under test (DUT) in a common emitter (source) configuration with a voltage supply and load inductor in the collector (drain) circuit. The DUT is turned on for a period of time sufficient to charge the inductor to the desired test current. The device is then turned off. The collapsing field in the inductor causes the collector voltage to rise to a level where the device may break down. As the voltage rises across the DUT, either the voltage will be limited by an external clamp imposed by the tester, allowing the device to safely turn off, or the device may begin to avalanche with or without entering normally destructive second breakdown. Second breakdown is characterized by a sudden collapse of voltage. If the DUT does experience second breakdown, it will be destroyed unless the current and voltage are removed very quickly. The success of making breakdown measurements is critically dependent on the speed of diverting current away from the DUT after the onset of second breakdown. The automated tester incorporates a fast breakdown detector and shunting “crowbar” circuit that diverts up to 100 A of test current from the DUT within 65 ns of device voltage collapse. The time to divert the current includes both a circuit propagation delay and current fall time and decreases to about 30 ns for test currents under 40 A. The voltage slew rate of the crowbar is 200 V/ns. The maximum test voltage is 2000 V, and the clamp voltage can be set at any level up to this maximum voltage.

The DUT base (gate) drive circuits are both constant current sources that can source and sink up to 25.5 A each for device turn-on and turn-off, respectively. A settable drive clamping circuit allows voltage limits to be imposed to prevent base-emitter breakdown and provides voltage drive when testing MOS gated devices.

Although the specific details and performance capabilities of the various earlier testers differ from those that characterize this tester, most of the testers have the same basic building blocks as those described up to this point. To automate the reverse-bias test, some additions and refinements over manually operated testers are needed. The most important improvements that must be made involve the protection circuit that detects second breakdown and diverts current from the DUT once breakdown occurs.

### 2.1 Breakdown Detector

**Figure 3:**
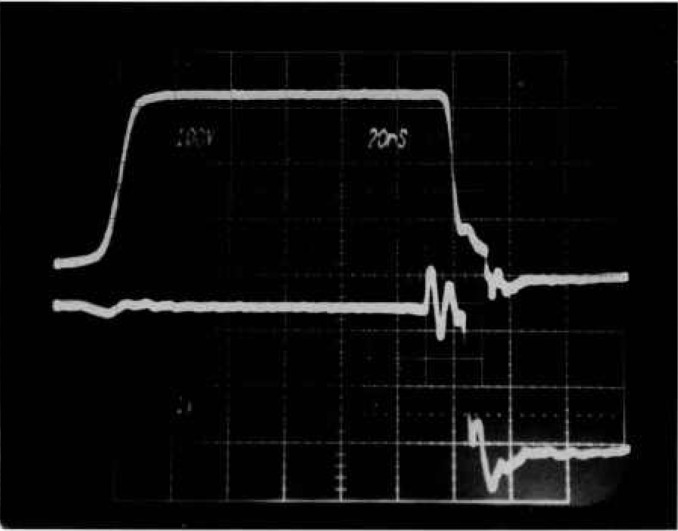
Voltage and current waveforms for sustained avalanche with second breakdown in MOSFET. Top trace: 100 V per small div.; bottom trace: 10 A per small div.; time: 20 ns per small div.

Presently, two methods are commonly used for nondestructive reverse-bias testing. One method uses a d*V*/d*t* detector that senses voltage collapse with a small capacitor which is coupled to an amplifier, which in turn drives a crowbar switch that shunts the current around the DUT. The other method is a pre-trigger scheme which always fires a crowbar during the test for breakdown. This scheme requires multiple tests whereby the trigger delay is adjusted in small increments until breakdown is observed. The first method is faster, in that multiple tests are not required to determine the presence of breakdown, and the second method is less demanding of the speed of the crowbar circuit. Figures 2 and 3 are oscillographs of device voltage and current waveforms taken by using the new tester that demonstrate problems with the above methods when automation is considered.

Figure 2a shows voltage and current waveforms when a 500-V power MOSFET is turned off very fast. A severe voltage overshoot reaching a peak of 480 V can be seen even though the clamp voltage was set to 180 V. However, no breakdown has occurred. The overshoot is caused by the parasitic inductance and diode turn-on delay in the clamping circuit in the presence of high d*I*/d*t*. Figure 2b shows voltage and current waveforms for actual second breakdown in a 200 V MOSFET. A d*V*/d*t* detector that is adequately sensitive to detect the collapse of voltage as second breakdown in figure 2b will be triggered falsely by the overshoot in figure 2a where there was no breakdown. False indications of breakdown are clearly unacceptable when automating the test for reverse-bias safe-operating area.

Figure 3 shows voltage and current waveforms for a power MOSFET that sustains in avalanche for 220 ns before entering second breakdown. Automating a tester based on a pre-trigger scheme is very difficult for delayed breakdowns because subjective interpretation is needed to determine if the voltage collapse is a result of second breakdown or of the crowbar firing. A further problem is that the length of time that a device stays in the avalanche mode is often subject to time jitter from test to test.

**Figure 4:**
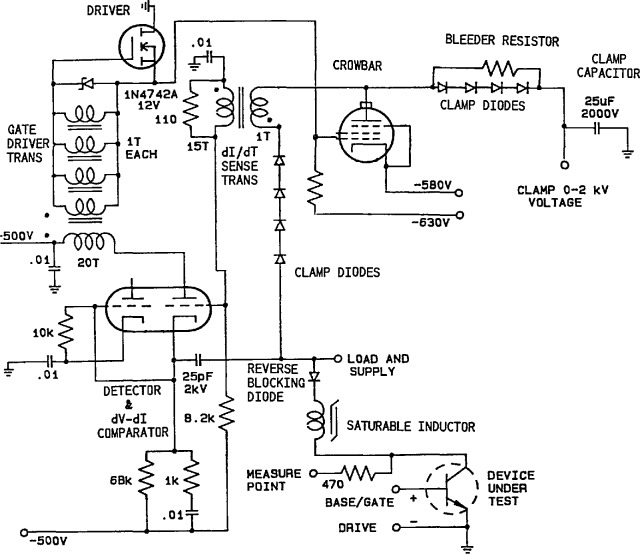
Simplified schematic showing critical elements of breakdown detector and crowbar circuit.

A unique breakdown detector circuit was therefore developed that uses both voltage and current to determine the presence of second breakdown. Figure 4 is a simplified schematic of the breakdown detection and protection crowbar. Half of a dual triode is used as a diode detector, and the other half is used as a comparator. The (+) input of the comparator (cathode) is pulled negative upon DUT voltage collapse, thus pulling the output plate negative and firing the crowbar unless the (−) input (grid) is driven negative by output from a dI/dt sense transformer that determines the presense of increasing clamp current. Increasing clamp current thus blocks the firing. Vacuum tubes are used because of their inherent high-voltage capability and low interelectrode capacitance. Tubes are free of recovery problems, do not need overvoltage protection, and are unsurpassed in speed.

### 2.2 Crowbar Performance Enhancement Techniques

Figure 4 also shows some additional important features. There are two sets of clamp diodes with the crowbar placed between them. During testing, the diodes nearest the DUT are reverse-biased to the maximum extent possible to keep parasitic capacitance at the test fixture low. A large negative voltage is applied to the crowbar cathode to increase the speed of current diversion from the DUT. A reverse blocking diode and saturable inductor work together to reduce current reversal in the DUT when the crowbar fires. The actual crowbar circuit utilizes 16 vacuum tubes connected in parallel that conduct the current for several hundred nanoseconds, after which SCRs (not shown) take over the crowbar function. The DUT voltage is measured through a 470 Ω resistor to reduce parasitic capacitive loading. This resistor causes a minor degradation to the bandwidth of the voltage measuring system.

### 2.3 Device Test Current and Test Load

In manually operated testers, a test current is usually set by running a series of tests with the clamp voltage set sufficiently low so as to prevent breakdown. The current is observed on an oscilloscope and increased or decreased to the desired level by adjusting the on-time or supply voltage. A desirable feature for automating the reverse-bias, safe-operating-area test is to have the ability to use the DUT current as an independent variable. Test current is determined by a number of factors, including the duration of the time that the DUT is turned on, the effective on-resistance of the device, resistances in the load circuit, and supply voltage. This tester incorporates a current limit detector which is coupled to the on-time generator. During either manual or automated tests, the on-time can be set to its maximum value, and as the DUT current ramps up and reaches a desired current set point, the on-time is terminated, the test is executed, and the new value of on-time is stored for subsequent tests.

**Figure 5:**
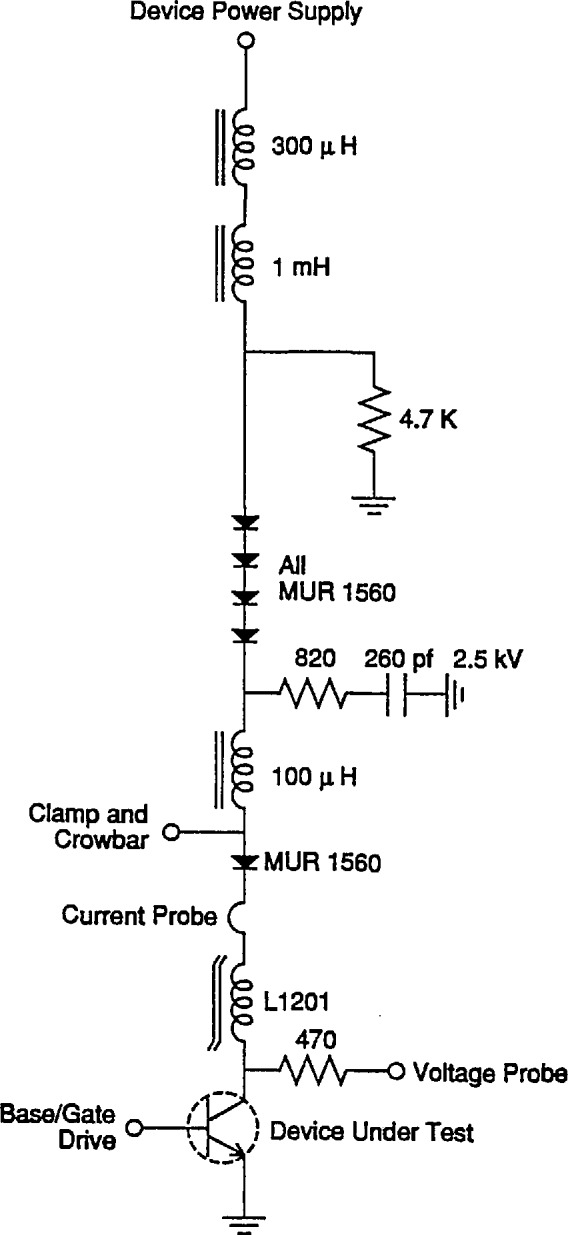
Load circuit for the Device Under Test.

While in principle the load for the DUT is simply an inductor, the use of several inductors with different values and saturation characteristics placed in series permits automated measurements over a wider range of currents. Figure 5 shows the device load used in the tester. The 100 μH inductor is linear up to the full 100 A test-current capability of the tester. The 300 μH inductor saturates at 15 A, and the 1 mH inductor saturates at slightly less than 1 A.

The 1 mH inductor works in conjunction with the four diodes and the 260 pF capacitor to prevent the voltage on the DUT from rapidly snapping to zero when the current in the 100 μH inductor goes to zero. Such a rapid voltage transition would otherwise be detected as a device breakdown. The resistors associated with this L-C-diode network are used for damping. The 1 mH inductor needs to store only enough energy to assure that the 260 pF capacitor is left in a charged state when the current goes to zero. The effect of the 1 mH inductor on the breakdown test itself is of no consequence, as it is in saturation for all test currents of interest, and it only adds a delay in the ramping up of the current when the device turns on.

The effective load inductance for test currents is 400 μH for currents up to 15 A, and about 100 μH for currents between 15 and 100 A. The dual-inductor system enhances the accuracy of the current-limit circuit at the lower currents because d*I*/d*t* is reduced, and additional time resolution is provided to establish the proper on-time pulse width needed to achieve the desired set current.

### 2.4 Voltage Clamp Power Supply

A large clamp capacitor is needed to effectively clamp large currents while maintaining a nearly constant voltage. During the course of making automated measurements, it is desirable to change the clamp voltage as fast as possible, and a power supply is needed that can both source and sink a significant amount of power. During repetitive testing, the clamp current charges the clamp capacitor, and this charge must be removed. A two-quadrant switching amplifier that sources and sinks up to 2000 V was developed to meet these requirements.

The switching amplifier can deliver a power output of up to ±60 W, or ±30 mA at up to 2000 V. Negative power represents power absorbed by the amplifier (negative current, positive voltage), and most of this power is not dissipated as heat, but converted back to the rectified power mains. A simplified schematic of the amplifier is given in figure 6a. The amplifier is configured as a rectified voltage source in series with a constant current sink, with the output taken between these and applied to a low-inductance 25 μF oil-filled capacitor. The capacitor is located as close to the DUT as possible.

The voltage-source portion of the amplifier is a quasi-resonant converter, a topology chosen to offer both the advantage of the wide control range of pulse-width modulation (PWM), and the inherent current limiting of the parallel resonant converter. The PWM circuit is driven by a feedback signal that includes the output of the amplifier and the output of a controlling DAC. The current-sinking portion of the amplifier is a parallel resonant converter operating at resonance. The ac feedback keeps the circuit self-oscillating at resonance over the wide range of supply voltage at the clamp capacitor. The interesting property of this circuit is that on a dc basis it behaves like a constant current sink over a wide range of supply voltage. The output current of this converter is fed back to the rectified power mains that runs the entire tester through a suitable transformer and rectifier, and is voltage-clamped by this rectified mains voltage. The output current returned to the rectified power mains is proportional to the clamp voltage. The operating frequency is about 110 kHz.

Figure 6b shows the output of the clamp supply when the tester makes a test at 2000 V. Once a test command is given, the clamp supply is gated on by changing the data fed to the DAC from zero to the desired test value. A time delay allows the voltage on the clamp capacitor to reach the set point before the breakdown test. Once the test is executed, the voltage returns to zero.

### 2.5 Tester Architecture

The functions in the tester that are programmable include test current, on-time, turn-on drive current, turn-off drive current, clamp voltage, and test-start. The tester can return a “device failed” message. The first five parameters above are represented as 8 bit binary numbers in the tester, and can be set either remotely through an IEEE 488 interface, or on the tester with rotary optical encoders. An encoder and 7-segment-type display are provided for each parameter. The interface uses the Fairchild 96LS488 chip^1^ which can be used in non-microprocessor, asynchronous systems such as this tester.

## 3. Breakdown Measurements

The measurement of the second breakdown voltage for a transistor can be done in two different ways, which can give two different numbers. One method is the unclamped measurement, whereby the clamp voltage is set well above the expected breakdown voltage. When the test is executed, the peak voltage at the point of voltage collapse is measured with a storage oscilloscope or a fast digitizer. The other method is the clamped measurement, whereby testing is begun by setting the clamp voltage well below the expected breakdown voltage, and incrementally raising the clamp voltage until second breakdown occurs. The clamp voltage is then equal to the breakdown voltage. The unclamped method generally gives a higher breakdown voltage indication than the clamped method. The unclamped method can give an artificially high number because a transistor can often withstand a higher voltage for a very short period of time before the voltage actually collapses. The clamped method can give an artificially low number because clamp overshoot can trigger second breakdown.

Figures 7a and 7b demonstrate the differences in the two measurement methods. In figure 7a, three different clamp settings cause three different responses for a bipolar transistor. For this figure, the transistor was turned off very hard, with a turn-off reverse base current of 4.8 A for a collector current of 6 A. For one trace, the clamp voltage was set well above the peak voltage recorded, which was about 640 V. Another trace was generated with the clamp set at 410 V. The voltage reached a peak value of about 510 V and the transistor voltage collapsed, but rather slowly compared to the unclamped case. A third trace was generated with the clamp set to 400 V, and the transistor turned off successfully, with the voltage reaching a peak of about 500 V. In figure 7b, the same transistor was tested with the same test conditions as in figure 7a, except that the turn-off current was reduced to a much more appropriate value of 1.0 A. Again, the first test was unclamped, with a peak recorded voltage of 550 V. When the clamp was set to 500 V, the transistor broke down with a peak voltage of about 510 V. With the clamp set to 490 V, the transistor did not break down and the voltage reached approximately 500 V.

In view of the above measurements, it is clear that some care must be taken when determining the second-breakdown, safe-operating area (SOA). Clearly, clamped measurements give a more conservative (lower voltage) SOA than unclamped ones, but clamped measurements can be overly conservative if the device is turned off too fast. The most accurate SOA is determined when the two methods are combined, by automating this tester with a programmable fast digitizer. To combine the methods, the clamp is raised incrementally until second breakdown occurs, and at the same time, the voltage waveform is digitized and the peak voltage reached is recorded for the breakdown.

**Figure 8:**
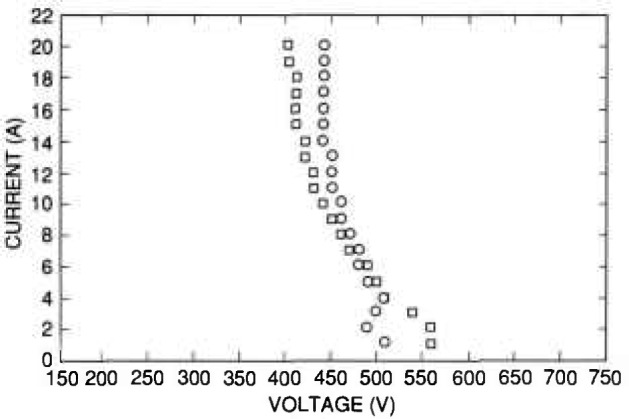
SOA curves measured by the tester under computer control for a bipolar transistor. The squares represent the SOA limit when the turn-on currents and turn-off currents are set to 1/5 the value of the collector current. The circles represent the SOA limit when the same transistor is tested with a fixed turn-on current of 2.0 A and a fixed turn-off current of 0.5 A.

Figure 8 gives some typical SOA curves for a bipolar transistor as measured by the tester under computer control. The measurements were made using the clamped technique without a digitizer, and the turn-off currents used were sufficiently low so as to avoid significant overshoots. One set of data was generated when the tester was programmed to make a series of tests with collector currents from 1 to 20 A, and the turn-on and turn-off base currents were adjusted for each collector current so that they were 1/5 the value of the collector current for turn-on and turn-off gains of 5. The other set of data was generated when the tester was programmed to run the tests for the same collector currents as previously used, but the turn-on and turn-off currents were maintained at the fixed values of 2.0 and 0.5 A, respectively. The data of figure 8 follow a general trend observed for bipolar transistors in that second breakdown occurs at lower voltages for higher collector currents, and also at lower voltages when higher turn-off currents are used.

**Figure 9:**
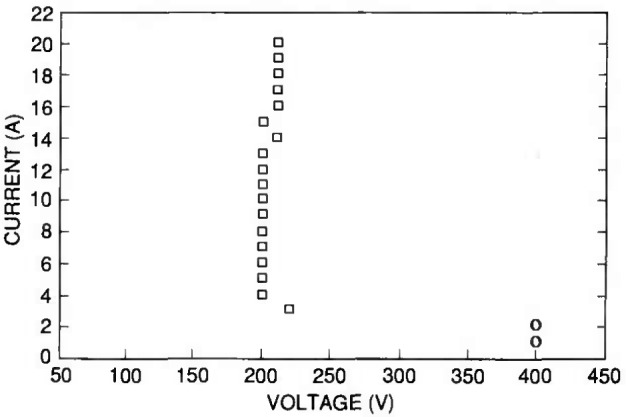
The squares represent the SOA curve for a power MOSFET. The circles at the lowest test currents indicate that the transistor did not enter second breakdown when the clamp voltage was set to 400 V, but rather sustained avalanche at a lower voltage.

Second breakdown data are given in figure 9 for a MOSFET. These data are representative of the SOA of MOSFETs, showing a nearly constant second breakdown voltage with drain current. For low currents, it is common for a device to avalanche without entering second breakdown as this device does. The data points represented as circles on the graph indicate that the device sustained avalanche without entering second breakdown when the clamp was set to 400 V (this voltage is not an indication of the actual voltage of the avalanche).

## 4. Limits On Nondestructive Testability

**Figure 10:**
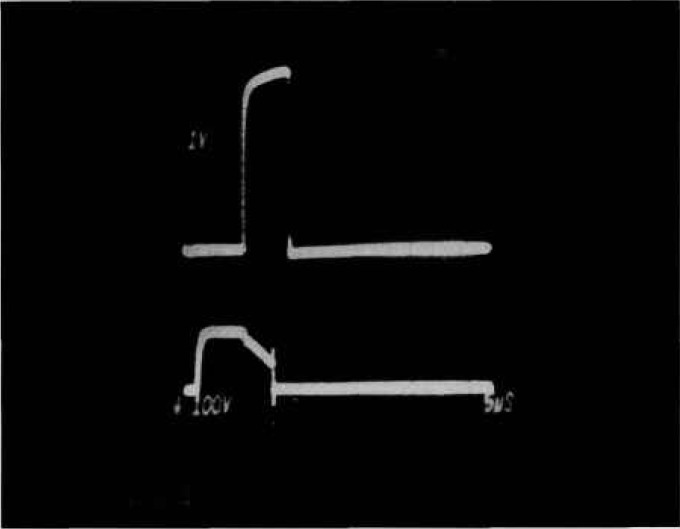
Voltage and current waveforms for a transistor that sustains avalanche for a relatively long time before entering second breakdown. Such behavior is often destructive, even when a protection circuit removes power very quickly. Top trace: 100 V per small div.; bottom trace: 10 A per small div.; time: 5 μs per small div.

Some transistors avalanche for a relatively long period of time before entering second breakdown, and thus absorb much more energy than transistors that break down without much delay. When transistors sustain avalanches for microseconds, they are often degraded or destroyed by the reverse-bias SOA test. Figure 10 shows typical voltage (top) and current waveforms that are generated by such a transistor during the reverse-bias second breakdown test. The voltage across the device rises when it begins to turn off, but begins to level off as the device avalanches. No clamp is acting. The avalanche voltage rises as the device heats internally. The current ramps down during the avalanche period because the load inductor maintains a higher voltage on the node nearest the device. At some point the device enters second breakdown, and the current is brought to zero by the protection circuit.

Bipolar transistors can usually survive a long sustaining period, but MOS-type devices are generally degraded or destroyed. Past experience has always indicated that the success with which devices are saved is strongly dependent on the speed of the protection crowbar circuit. Although this automated tester has an extremely fast crowbar, it is not able to save many of the devices that have long sustain times. The question arises as to whether these devices could be saved if the protection were even faster.

There are also other possible stresses on the device when it enters second breakdown. The localized portion of the device where second breakdown is instigated absorbs additional energy during voltage collapse because the parasitic capacitance of both the test fixture and the device itself is discharged through the breakdown site. The total parasitic capacitance of the test fixture in the automated tester is 83 pF, and internal device capacitances can be substantially larger. Once the crowbar circuit acts, there is some reversal of current in the device, although this automated tester keeps it to a minimum with the saturable reactor and reverse-blocking diode mentioned previously. It is possible that these additional stresses are responsible in part for the destruction of these devices.

A further attempt was made to nondestructively test these devices by constructing another tester. Reliability and ease of use concerns were set aside to make the fastest device protection possible. In this special tester, the load inductor and crowbar current shunt are eliminated and replaced with a controlled current source that can be turned off quickly. The controlled current source is driven in such a way as to simulate the load inductor in the traditional reverse-bias SOA test. The waveforms shown in figure 10 were made by using this special tester. Additional goals achieved with this special tester were reduced test-fixture capacitance and no current reversal in the device when the protection acts. The parasitic test-fixture capacitance is 62 pF, and there is no clamp provision. This special tester can test at voltage and current levels of up to about 1600 V and 25 A, respectively.

**Figure 11:**
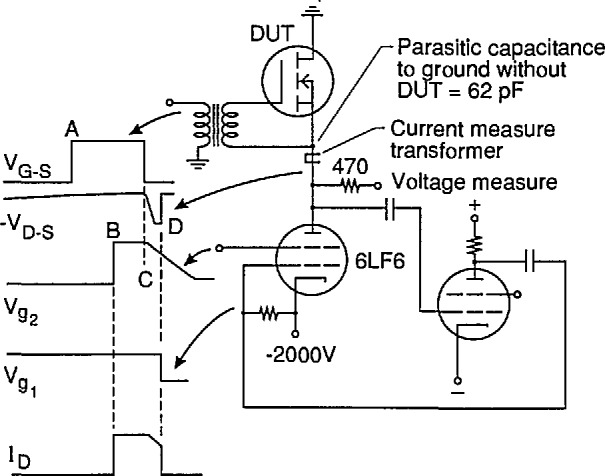
Simplified schematic of special reverse-bias SOA tester that was developed for extremely short device protection times. Timing waveforms for operation are indicated and are as follows: A - Device gate is turned on B - Constant current source is turned on C - Device is turned off and current source ramp-down begins D - Device breaks down, and current source is turned off fast.

Figure 11 is a simplified schematic of the critical portion of this tester. Representative waveforms that govern the circuit’s operation are also shown. A single beam-type pentode acts as a current source and replaces the inductor in the traditional test. The current source is in series with the DUT. However, the source is located on the negative voltage side of the DUT, such as the source terminal of an n-channel FET as shown in the figure. Location of the current source in the drain circuit would lead to higher parasitic capacitance at the test fixture and would complicate the driver circuits for the pentode. During the test, the gate of the DUT is turned on first. The current-source pentode is then turned on with a positive-going step function with an amplitude that determines the desired test current. This step function, which can have an amplitude of up to 1500 V at 2 A (with respect to the cathode) for the highest test currents, is applied to grid 2. Grid 1 is maintained at 0 V during this phase of the test. These conditions are maintained for about 10 μs to give the charge distribution and carriers in the DUT time to reach equilibrium. The DUT is then turned off, and the voltage across the device rises. At the same time, the step function applied to grid 2 begins a linear ramp down with a slope that can be adjusted to simulate inductors of different sizes. If the simulated inductor is set to a sufficiently small value, or the test current is sufficiently low, the DUT may simply avalanche until the current goes to zero. If the DUT experiences second breakdown, the voltage collapses, and the collapse is sensed as a fast d*V*/d*t* signal and is used to drive grid 1 of the pentode negative to turn it off. The current source is thus open-circuited, and there cannot be any current reversal in the DUT as there is with the crowbar that depends on the recovery of a diode.

**Figure 12:**
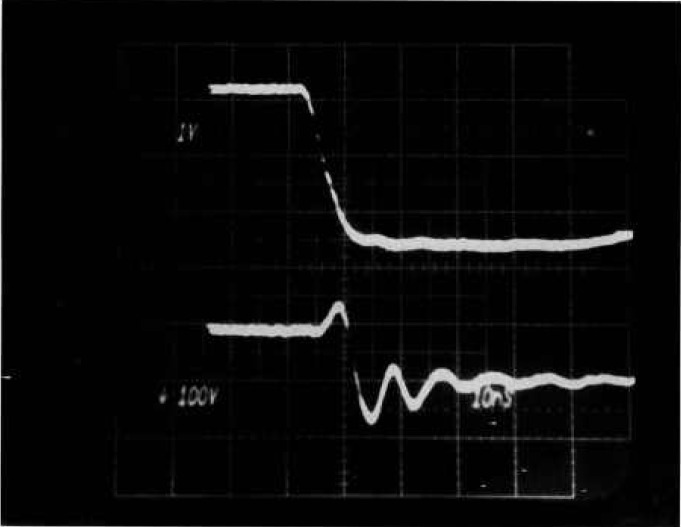
Voltage and current waveforms for second breakdown in a MOSFET as observed with the special tester. The current is removed in less than 10 ns after breakdown. Top trace: 100 V per small div.; bottom trace: 10 A per small div.; time: 10 ns per small div.

The protection in this tester is extremely fast, as demonstrated by the waveforms in figure 12. Shown are voltage (top) and current waveforms for a MOSFET second breakdown. The test current was 20 A and the breakdown voltage was 550 V. The time scale was 10 ns, and the current was brought to zero within 10 ns of voltage collapse. Voltage collapse corresponds to the upturn in current just before it was brought down. The upturn is caused by discharge of the test-fixture parasitic capacitance. The voltage waveform does not give a good time reference for breakdown because of substantial time jitter from test to test on this fast time scale, and two tests are needed to capture the two waveforms. The voltage transistion was actually faster than it appears in the figure because of bandwidth limiting caused by the 470 Ω isolation resistor. The current was measured with a Pearson 411 current-to-voltage transformer, and the speed of the measurement system may be a limiting factor in determining the actual speed of the protection.

As fast as this special reverse-bias SOA tester is, it is not able to consistently save the devices that sustain long avalanches which cannot be saved by the automated tester. It is perhaps less destructive because some devices that can survive only one or two tests on the automated tester can survive three or four on this special tester before being degraded. It is possible that devices that do sustain for long periods of time before entering second breakdown actually are degraded by localized high temperatures before actual voltage collapse.

## 5. Conclusions

An overview for the design of an automated reverse-bias safe operating area tester has been given, with emphasis on special circuits that permit automation. Some measurement examples have been discussed, as well as sources of error in the measurements. The reverse-bias second breakdown measurement is generally nondestructive, provided current and voltage in the device under test are removed very quickly once second breakdown occurs. Some devices that sustain avalanches for relatively long times (microseconds) before entering second breakdown are not saved by even the fastest of protection circuits.

## Figures and Tables

**Figure 2a:**
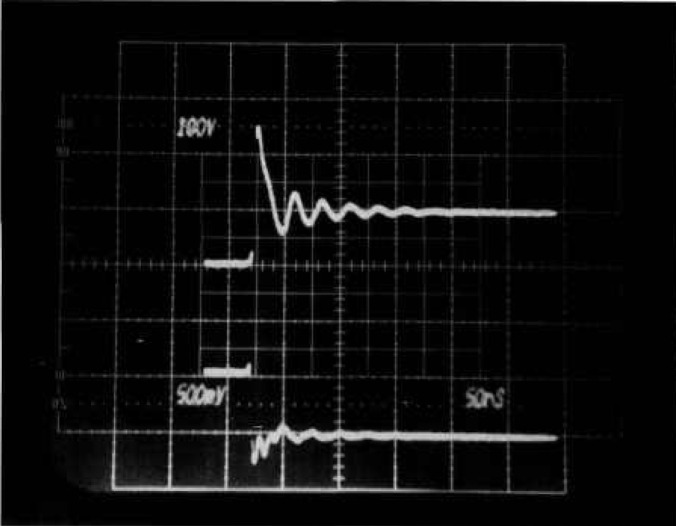
Voltage and current waveforms showing voltage overshoot for fast MOSFET turn-off. Top trace: 100 V per small div.; bottom trace: 5 A per small div; time: 50 ns per small div.

**Figure 2b:**
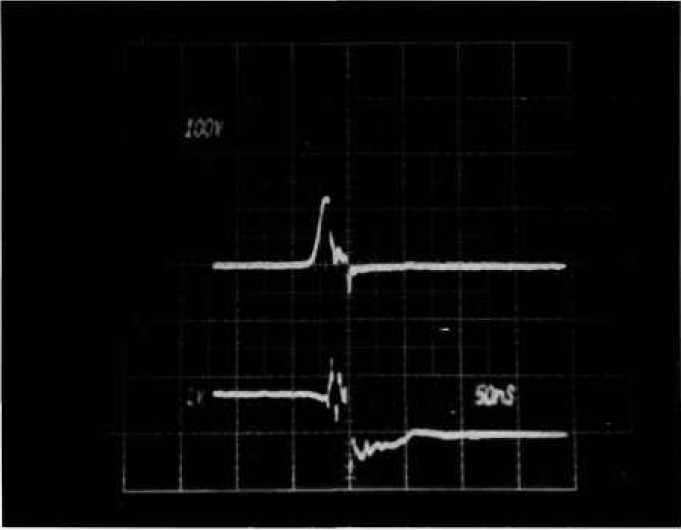
Voltage and current waveforms for second breakdown in 200V MOSFET. Top trace: 100 V per small div.; bottom trace: 10 A per small div.; time: 50 ns per small div.

**Figure 6a:**
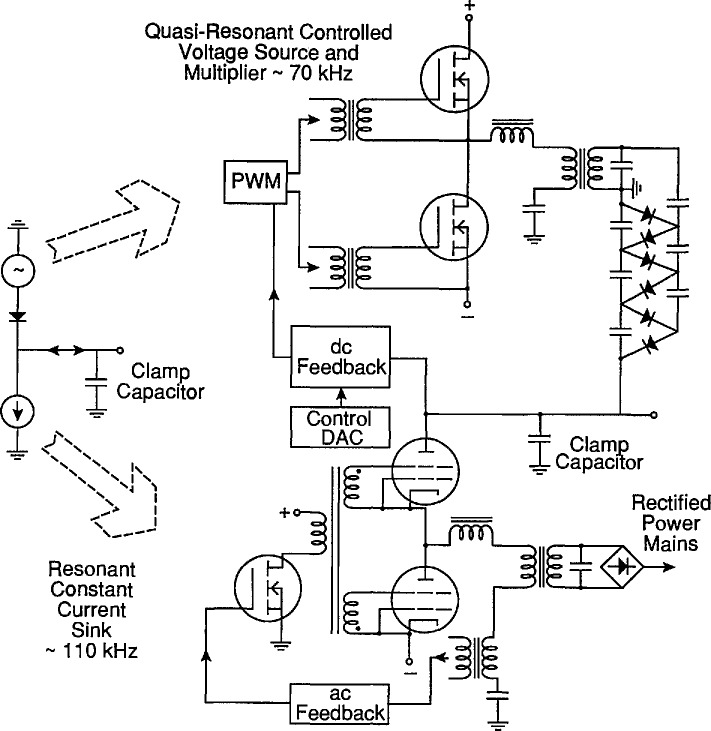
Simplified schematic of two-quadrant switching amplifier for clamp supply. A conceptual circuit appears to the left, and the actual implementation is shown on the right.

**Figure 6b:**
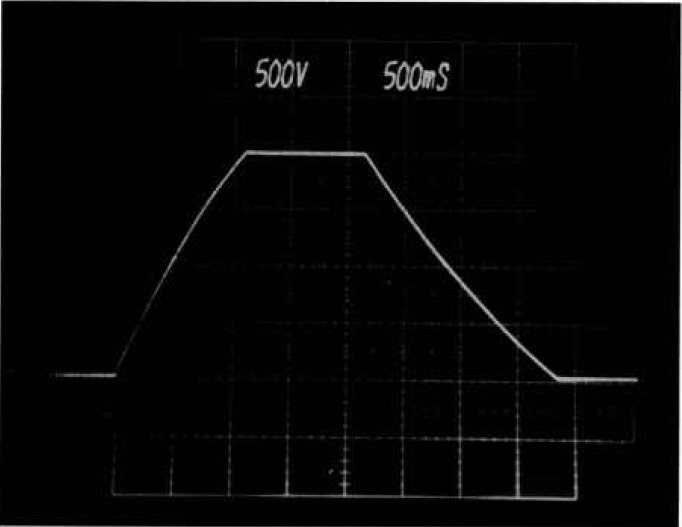
Clamp voltage for a test at 2000 V. Once a test command is given, voltage on the clamp capacitor ramps up to the desired clamp voltage, the test is executed, and the voltage returns to zero. Scale: 500 V per div.; time: 500 ms per div.

**Figure 7a:**
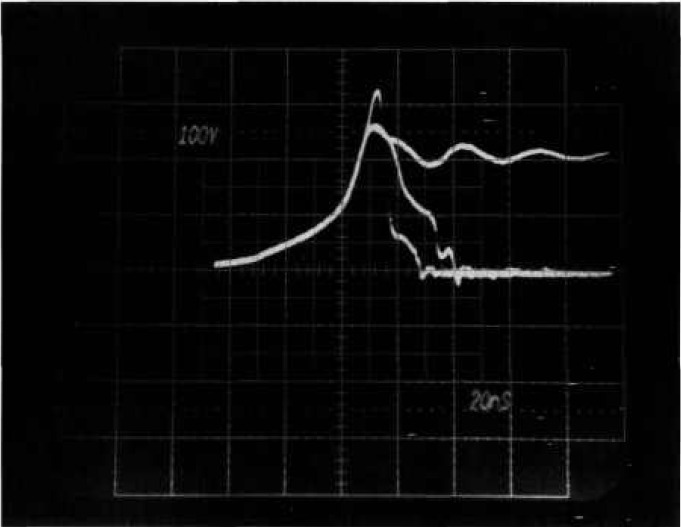
Device collector voltage for three different clamp settings for high turn-off base current. The transistor breaks down for two of the clamp settings. Scale: 100 V per small div; time: 20 ns per small div.

**Figure 7b:**
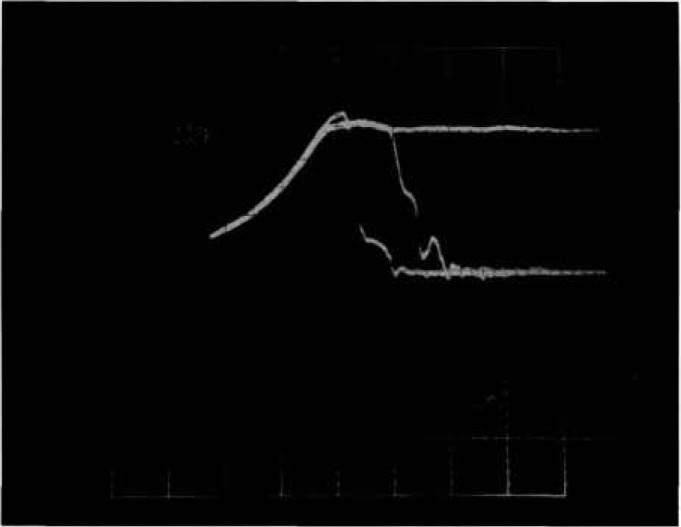
Same device and conditions as above; however, the turn-off is reduced, and a different set of clamp voltages is used. The voltage difference between unclamped and clamped measurements is reduced for reduced turn-off drive.

## Footnotes

1 Certain commercial equipment, instruments, or materials are identified in this paper to specify adequately the experimental procedure. Such identification does not imply recommendation or endorsement by the National Institute of Standards and Technology, nor does it imply that the materials or equipment identified are necessarily the best available for the purpose.
